# Could mycotoxigenic *Fusarium* sp. play a role in ulcerative dermal necrosis (UDN) of brown trout (*Salmo trutta* morpha *trutta*)?

**DOI:** 10.1007/s12550-020-00395-8

**Published:** 2020-05-05

**Authors:** Agnieszka Pękala-Safińska, Piotr Jedziniak, Anna Kycko, Mateusz Ciepliński, Ewa Paździor, Łukasz Panasiuk, Mariusz Kasprzak, Leszek Jerzak

**Affiliations:** 1 Department of Fish Diseases, National Veterinary Research Institute, Al. Partyzantów 57, 24-100 Puławy, Poland; 2 Department of Pharmacology and Toxicology, National Veterinary Research Institute, Al. Partyzantów 57, 24-100 Puławy, Poland; 3 Department of Pathology, National Veterinary Research Institute, Al. Partyzantów 57, 24-100 Puławy, Poland; 4 Department of Zoology, Faculty of Biological Sciences, University of Zielona Gora, ul. Prof. Z. Szafrana 1, 65-516 Zielona Gora, Poland; 5 Department of Nature Protection, Faculty of Biological Sciences, University of Zielona Gora, ul. Prof. Z. Szafrana 1, 65-516 Zielona Gora, Poland

**Keywords:** Fungi, Mycotoxin, Zearalenone, Ulcerative dermal necrosis (UDN), Fish diseases

## Abstract

*Fusarium* infections have been reported in aquatic animals, but are still poorly investigated in wild salmonids. The aim of the study was to determine the impact of the fungi and their toxins on the health status of brown trout (*Salmo trutta* morpha *trutta*) migrating from the Baltic Sea to the freshwater. Individuals from the wild brown trout population exhibiting ulcerative skin lesions were collected from the Słupia River in Poland and subjected to microbiological, histopathological, and hematological examinations, as well as toxicological analysis for a presence of mycotoxins. The results of microflora isolation from the brown trout skin samples revealed the presence of conditionally pathogenic bacteria and fungi classified by molecular techniques as *Fusarium* spp. Toxicological analysis allowed for detection of zearalenone (ZEN) in the liver, kidney, and gastrointestinal tract of the fish. In several cases, there was α-zearalenone (α-ZEL) identified at trace levels in the liver, as well as sterigmatocystin and enniatin B at low levels in the kidney and the liver. Histopathological examination revealed the presence of fungal hyphae disrupting the epidermis and penetrating into the necrotic dermis and hypodermis. The decreased values of the blood parameters, i.e., hemoglobin concentration (HGB), packed cell volume (PCV), mean corpuscular volume (MCV), mean corpuscular hemoglobin (MCH), and white blood cell count (WBC), were indicative of osmoregulation failure being a consequence of the skin damage. The results of the study provide new information regarding *Fusarium* sp*.* infection in brown trout and serve as the basis for further research on the potential impact of the fungi and their mycotoxins on the Baltic salmonid population, including their role in ulcerative dermal necrosis.

**Electronic supplementary material:**

The online version of this article (10.1007/s12550-020-00395-8) contains supplementary material, which is available to authorized users.

## Introduction

*Fusarium* is a genus of fungi which belong to the phylum *Ascomycota*, class *Sordariomycetes* (Buller 2014). They are known as ubiquitous organisms widely distributed throughout the world, both in temperate and in tropical regions. While many species are considered to be plant pathogens (Gupta et al. 2000; Jeschke et al. 1990), some of them are also commonly known as opportunistic pathogens for fish (Buller 2014). Until now only black gill disease was described as a disorder caused by different *Fusarium* species: *F*. *oxysporum* and *F*. *solani* in prawn *Penaeus japonicus* (Khoa and Hatai 2005; Khoa et al. 2005), *F*. *tabacinum* (in Atlantic stream crayfish *Austropotamobius pallipes*) (Alderman and Polglase 1985). Fungi of the genus *Fusarium* have also been recognized as potential producers of mycotoxins harmful to both humans and animals, acting directly when digested or inhaled, or indirectly through the consumption of contaminated feed (Chełkowski 1985). The following highly potent mycotoxins were described as produced by *Fusarium*: deoxynivalenol, nivalenol, moniliformin, ochratoxin A, and zearalenone (Fiedler et al. 2001; Pietsch et al. 2013; Schollenberger et al. 2007). The compounds above caused systemic disorders manifesting themselves by hepatotoxic, nephrotoxic, cardiotoxic, dermatotoxic, and neurotoxic effects. They also have been found to affect the hormonal balance and reduce immunity (Chełkowski 1985; Pietsch et al. 2015a). Meanwhile, there are still gaps of knowledge concerning the impact of *Fusarium* species and their toxins on the health status of fish, especially *Salmonidae*.

The clinical symptoms of fungal infection are quite characteristic. In the beginning, circular or crescent-shape skin lesions are present, developing rapidly and causing destruction of the epidermis (Willoughby 1989). In the recent year, similar skin disorders were observed in brown trout (*Salmo trutta* morpha *trutta*) flowing into the Polish rivers of the Baltic Sea for spawning. These symptoms were also reminiscent of ulcerative dermal necrosis (UDN), a disease of unexplained etiology (Murrphy 1973; Roberts 1972; Roberts et al. 1971).

The present study aimed to determine the cause of the health disorders of the brown trout migrating into Polish freshwater and a possible role of *Fusarium* spp. and their toxins in the fish mortality. Our results may also provide a new view on the etiology of UDN disease and revise the current approach to this disease.

## Materials and methods

### Sample collection

Wild individuals of brown trout from the freshwater Słupia River were collected for the laboratory examinations. Fish were caught straight into the net, in the natural fish ladder, which was a branch of this river, in the place where usually fishermen capture the brown trout to perform an artificial spawning. The fish were divided into three groups of thirty individuals each. The first group consisted of moribund brown trout exhibiting clinical symptoms of health disorders manifested by skin lesions. Samples of the skin, gills, and internal organs (the kidney, liver, spleen) were collected separately for bacteriology and mycology as well as toxicology and histopathology. For hematologic evaluation, additional two groups of wild brown trout were used: one group consisted of the healthy individuals and the second one involved fish showing visible skin lesions. Sex ratio in these two groups was 1:1. Blood was collected from the caudal vein and immediately transferred into a standard test tube containing K_2_EDTA anticoagulant.

### Bacteriological and mycological examination

Tissue samples of the skin, liver, and kidney were immersed in sterile phosphate-buffered saline (PBS) (Biomed, Lublin, Poland) in the ratio of 1:1 (w/v), homogenated, and then inoculated onto appropriate media. For bacteriological examinations, agar supplemented with 5% horse blood (BA) (Biomed, Lublin, Poland) and trypticase soy agar (TSA) (BioMérieux, Marcy l’Étoile, France) were used. Mycological studies were performed using Sabouraud agar (Biomaxima, Lublin, Poland). After inoculations, all the media were incubated at 27 °C ± 1 °C, 72–96 h for bacteriology and 5 days for mycology (Buller 2014).

The dominant types of bacterial colonies were re-isolated; then, pure cultures were used to assess their morphology as well as Gram staining. Biochemical identification was performed using API and VITEK2 system (BioMérieux, Marcy l’Étoile, France), according to the manufacturer’s instructions. In case of doubtful biochemical results, sequencing of 16S rRNA gene was carried out as described previously (Pękala et al. 2018).

The fungus culture on Sabouraud agar medium was carried out for 5 days at 27 °C, and the presence of hyphae in examined samples was studied. The fungal hyphae were then collected and inoculated onto Sabouraud liquid medium in order to isolate a total DNA with DNeasy Plant Mini Kit (Qiagen, Hilden, Germany). Conventional semi-nested PCR targeting conserved ribosomal internal transcribed spacer (ITS) region were performed as described previously (Ferrer et al. 2001). Amplified products (about 280 base pairs) were purified by USB ExoSAP-IT PCR Product Cleanup method (Affymetrix), sequenced using 3730xl DNA Analyzer (Genomed S.A.), and analyzed with the MEGA 5.05 software (Center for Evolutionary Functional Genomics, The Biodesign Institute, Tempe, USA).

### Histopathology examination

For histopathology, the following collected samples of brown trout were fixed in 10% neutral-buffered formalin: skin with muscles displaying the gross changes, gills, liver, kidney, and spleen. The samples were routinely processed, embedded in paraffin blocks, and cut on microtome at 4 μm. The cut sections were stained using hematoxylin-eosin method (HE) and examined using light microscopy for the presence of histopathological lesions.

### Toxicological examination

For toxicological analysis, LC-MS/MS technique was used to determine the presence of 25 mycotoxins (including aflatoxin B_1_, B_2_, G_1_, G_2_; deoxynivalenol; fumonisin B_1_, B_2_; ochratoxin A; toxin T-2 and HT-2; zearalenone (ZEN); alfa-zearalenone (α-ZEL); beta-zearalenone (β-ZEL); citrinin; nivalenol; fusarenon-X; diacetoxyscirpenol; sterigmatocystin and beauvericin; enniatin A, A_1_, B, B_1_) in the gastric contents, kidney, and liver. All standards were purchased from Sigma-Aldrich (Milan, Italy). All solvents and reagents were purchased from Avantor (Radnor, PA, USA). The method was a modification of our previously published procedure (Panasiuk et al. 2019). The homogenized samples (*n* = 10, 2 g of tissues and 1 g of gastrointestinal content, each sample was analyzed once) were extracted with a mixture of acetonitrile:water:acetic acid (79:20:1, v:v:v) and clean-up with solid-phase extraction (OASIS HLB cartridges, Waters, Etten-Leur, The Netherland). Finally, the sample was transferred to orange vials and determined with the LC-MS/MS technique (chromatograph Nexera X2 coupled with the tandem mass spectrometer LCMS 8050, Shimadzu, Kyoto, Japan), operated in positive and negative modes. The sample extract was analyzed using the following chromatographic conditions: mobile phase with NH4Ac and MeOH (pH ~ 3.4)–gradient elution and Kinetex Biphenyl column (100 × 2.1 mm, 2.6 μm; Phenomenex, Torrance, CA, USA). For all mycotoxins, at least two transitions (multiple reaction monitoring modes) were monitored in the tandem mass spectrometer. The qualitative analyses were conducted with matrix-matched calibration curve and the use of labeled internal standards. Characterized method performances (recoveries and precision) for all analytes were satisfactory, with a limit of detection (LOD) and quantification (LOQ) for most of the analytes at level 2 μg/kg were presented in the Supplementary Materials.

In the case of ZEN findings in the livers and kidney, the confirmatory analysis was performed. The sample preparation was performed with enzymatic hydrolysis: 1 g/mL of sample was digested with 50 μL β-glucuronidase type H-2 from *Helix pomatia* (Sigma-Aldrich, Milan, Italy) to hydrolyze glucuronide conjugates of mycotoxins and their metabolites, incubated in 37 °C overnight, then diluted with PBS (1: 2) and purified with combination of AOF and DZT column (R-Biopharm, Darmstadt, Germany)–multi-antibody immunoaffinity column: washed with 10 mL deionized water and eluted with 3 mL methanol. Eluent was dried under nitrogen stream at 45 °C and reconstituted in 100 μL mobile phase A and 100 μL mobile phase B. During the LC-MS/MS analysis, separation of analytes was carried out on a Luna Omega Polar column (3 μm, 2.0 × 150 mm; Phenomenex, Torrance, CA, USA) equipped with a C18 guard column (2 × 4.6 mm, ID; Phenomenex, Torrance, CA, USA). Eluent A was 95% MeOH (5% 10 mM ammonium acetate and 0.001% acetic acid in water) and eluent B was 95% 10 mM ammonium acetate and 0.001% acetic acid in water (5% MeOH) at a flow rate of 600 μL min^−1^ and the injection volume was 5 μL. The total runtime is 15 min. The gradient started at 100% B for 2 min. Solvent A was increased to 60% until 2 min and kept for 4.5 min at 60% A, then increased to 95% until 6 min and kept for 10 min at 95%. Afterwards, the percentage of A is decreased to starting conditions (10.1 min) and the column is allowed to re-equilibrate until 15 min. Detection of ZEN was carried out on an AB SCIEX QTRAP^®^ 6500 (Sciex, Concord, Ontario, Canada) mass spectrometer with ESI ionization in positive and negative ionization modes. ESI source parameters are optimized and present for all measurements as follows: source temperature, 350 °C; curtain gas, 35 psi; gas 1.60 psi; gas 2.35 psi. Ion spray voltage is set to − 4000 V in negative ionization mode. Two characteristic MRM transitions were monitored to ensure accurate identification. The limit of quantification (LOQ) for ZEN was at level 1 μg/kg.

### Hematological examination

Blood analysis was performed shortly after sample collection. Basic hematology parameters were investigated: packed cell volume (PCV), hemoglobin concentration (HGB), red blood cell count (RBC), mean corpuscular hemoglobin (MCH), mean corpuscular volume (MCV), mean corpuscular hemoglobin concentration (MCHC), white blood cell count (WBC). Laboratory analysis was conducted according to manual protocol previously described by Cieplinski et al. (2018). Drabkin’s reagent used in this investigation for determination of hemoglobin concentration was manufactured in Poland, Lodz, Lodzkie Province by Kolchem. Statistical analysis was used to show significant differences in mean blood indices between healthy and diseased fish. Due to lack of Gaussian distribution in presented parameters, the Mann–Whitney *U* test for independent groups was used. Statistica 12.5 software was used to perform statistical analysis (StatSoft 2006).

## Results

### Clinical symptoms

**Fig. 1:**
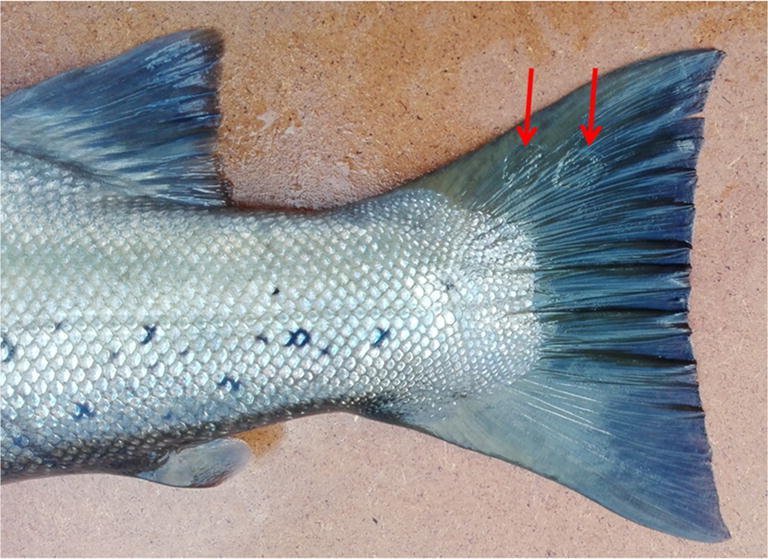
Small oval patches’ presence on the tail fins of brown trout

**Fig. 2:**
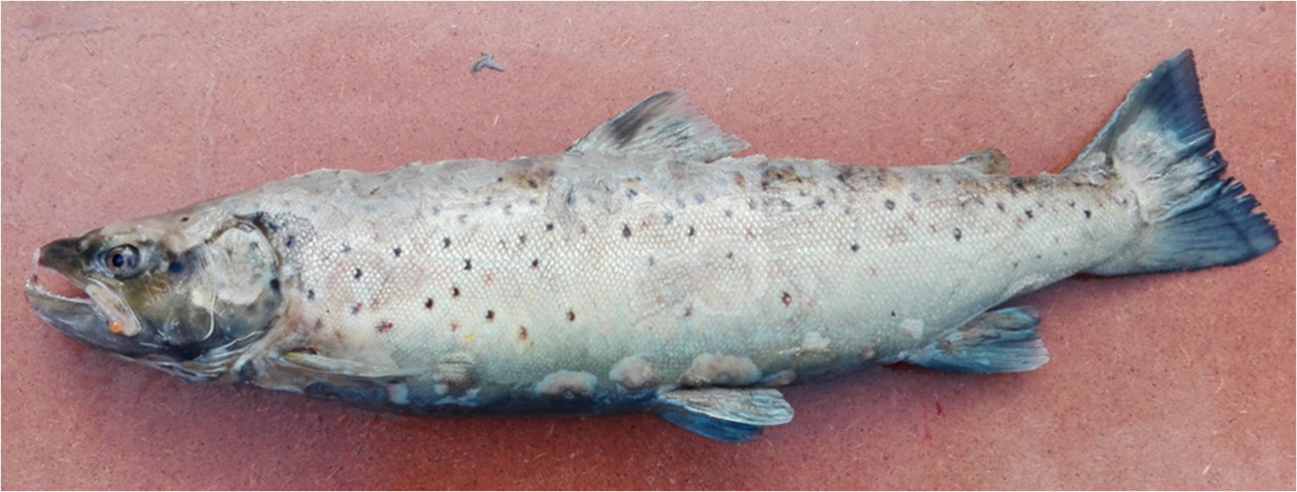
Skin depigmentation with focal lesions located all over the fish’s body

**Fig. 3:**
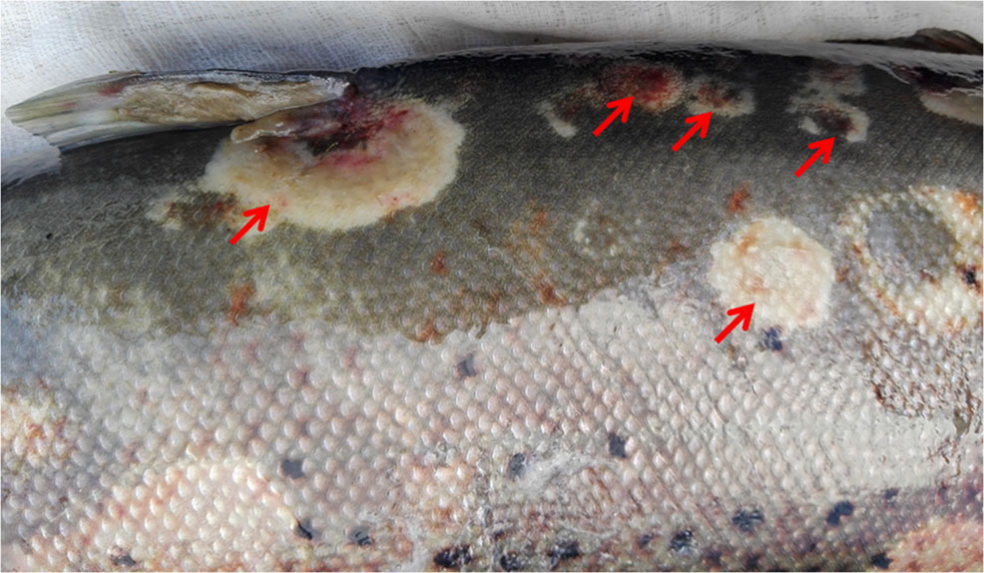
Focal radial or oval lesions located at the dorsal part of the fish’s body

Small oval patches were noticed on the tail fins of brown trout in the early stage of the disease (Fig. 1). These symptoms developed rapidly, spreading onto the almost entire body, manifesting themselves as focal, oval-shaped skin erosions with depigmentation and epidermal loss located mainly on the cranial and dorsal part of the body and leading to the fish mortality within 2/3 days (Figs. 2 and 3). Post-mortem examination showed no pathological symptoms and changes in the internal organs. This phenomenon concerned mainly the broodstock males.

### Bacteriological and mycological examination

Bacteriological studies of the skin lesion samples supported by molecular analysis revealed the following dominant species of bacteria: *Acinetobacter* spp., motile and mesophilic *Aeromonas* strains like *Aeromonas hydrophila* and *Aeromonas sobria*, *Chryseobacterium* spp., *Pseudomonas fluorescens*, *Serratia liquefaciens*, and *Shewanella* spp. From internal organs (the liver and kidney) *Aeromonas sobria* and *Shewanella putrefaciens* were mainly isolated. In several cases, no bacterial growth was observed.

**Fig. 4:**
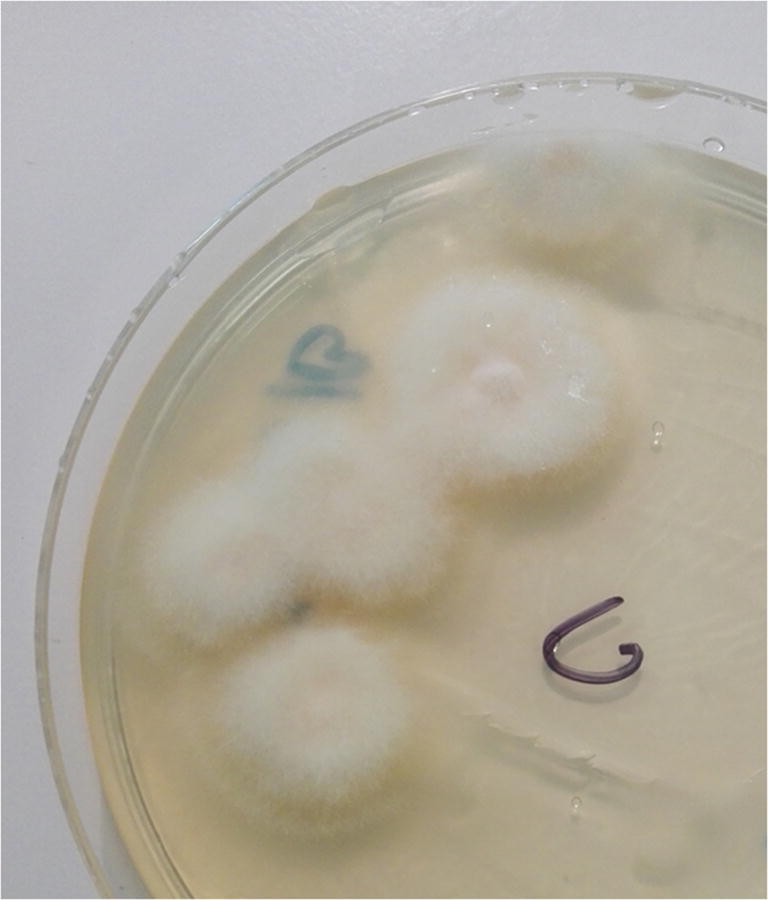
Cotton-like, white mycelium on Sabouraud medium

**Fig. 5:**
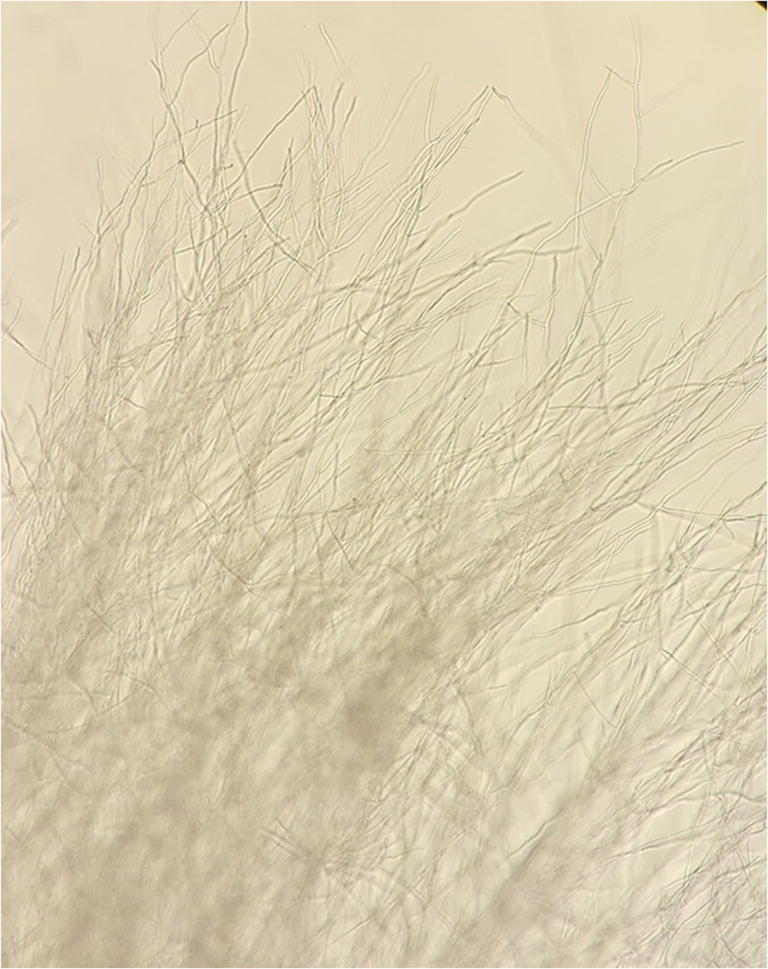
Segmented structure of fungal hyphae under microscopy examinations (× 50)

Mycological studies of the same skin samples revealed the growth of cotton-like, white mycelium on Sabouraud medium (Fig. 4), which showed a segmented structure of hyphae in the microscopic examination (Fig. 5). The results of the ITS region sequencing allowed to classify the fungus as *Fusarium* sp. with similarity to *Fusarium tricinctum* (KJ598871), *F*. *avenaceum* (MH299915), and *F*. *lateritium* (MF687693) at level 99.6%. Gene sequences of *Fusarium* sp. were deposited in the GenBank database under accession number MK789858.

### Histopathological examination

**Fig. 6:**
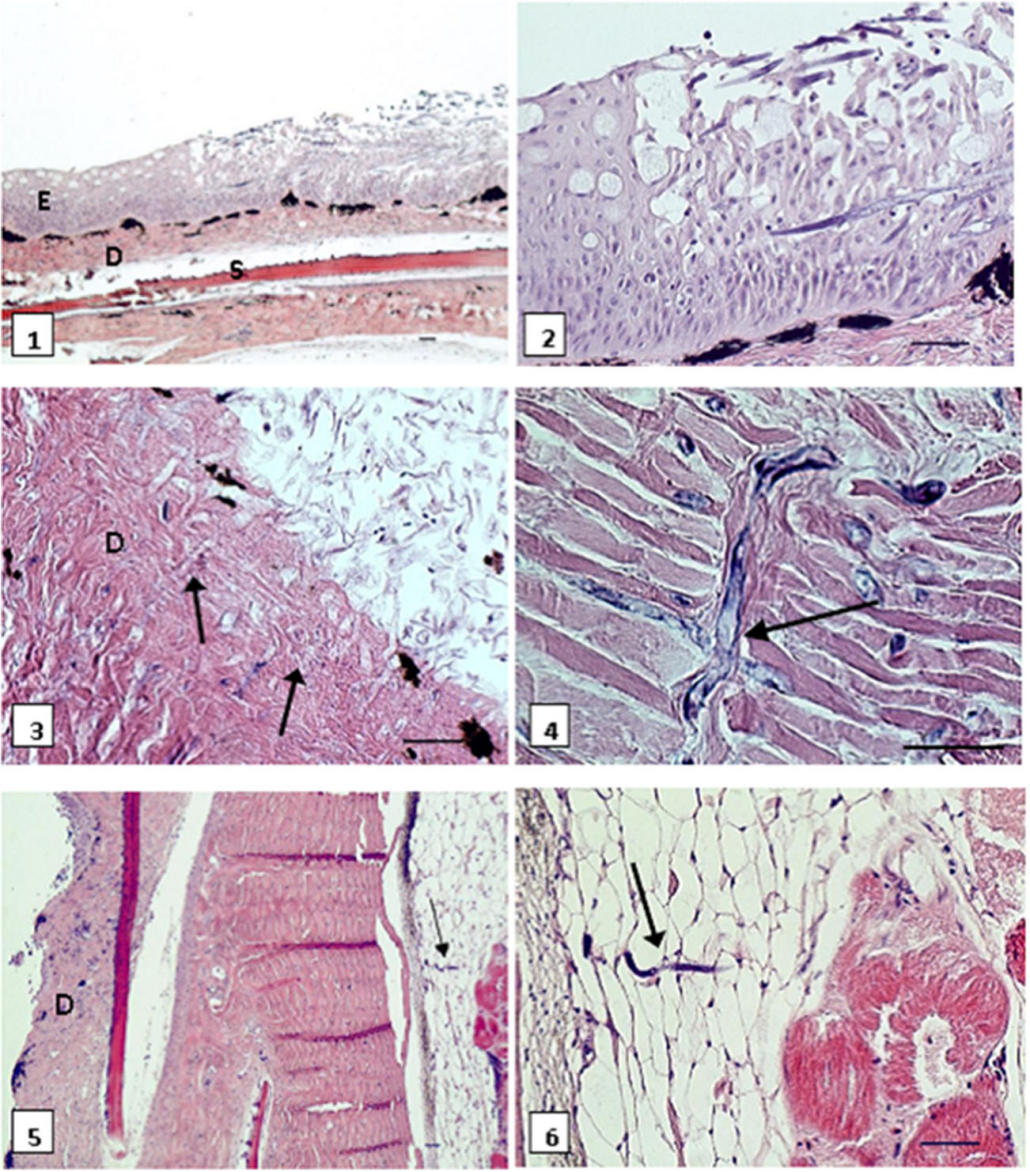
Brown trout, skin. (1) Epidermis affected by fungi (upper right). (2) Closer view of the fungal hyphae penetrating the epidermal layer. (3) Loss of epidermis, the fungal hyphae (arrows) invading necrotic dermis. (4) Fungal hyphae (arrow) in the dermal layer. (5) Loss of epidermis, mild inflammation in the dermis (left), fungal hyphae (arrow) penetrating into the hypodermal layer. (6) Higher magnification of the fungal hyphae in the hypodermis, extending to muscular layer (right). E, epidermis; D, dermis; S, scale. HE, bar = 50 μm

Histopathological examination of all the skin sections revealed lesions characterized by disruption and necrosis of the epidermal layer with the presence of light-to-dark basophilic fungal hyphae penetrating the epidermis, dermis, and hypodermis. The hyphae were measured 5 to 7 μm in diameter and were characterized by acute right angle branching. Partially, there was a complete loss of epidermis visible, accompanied by dermal necrosis, edema, and, occasionally, myofibrillar necrosis. In several cases, mild inflammatory infiltrations consisting of macrophages, lymphocytes, and single granulocytes were present, usually limited to eroded dermal layer (Fig. 6). In all the examined gills, moderate hyperemia of the vessels was observed, with occasional focal epithelial hyperplasia in primary lamellae. There were no noticeable changes found in any section of the liver, kidney, or spleen.

### Toxicological examination

Toxicological analysis revealed the presence of ZEN in the liver, the kidney, and the gastrointestinal tract at a level range 2–25 μg/kg. Moreover, α-ZEL was found in two livers, however only in trace amounts (below the limit of quantitation [LOQ] of the method). In at these two cases, sterigmatocystin and enniatin B were detected (in two kidneys and two livers), but at a low level, below 9 μg/kg. The mycotoxins were not found in the fish muscles above the limit of quantitation (Table 1), but trace amounts of mycotoxins were found in most of the samples.

**Table 1:** Mycotoxins found in the fish tissues and organs for the group with clinical symptoms

	Muscle (μg/kg)	Liver (μg/kg)	Kidney (μg/kg)	Gastrointestinal tract (μg/kg)
Zearalenone	< LOD	6.51–11.2	11.9–25.3	2.02–9.92
α-Zearalenone	< LOQ	< LOQ	< LOQ	< LOQ
Sterigmatocystin	< LOQ	0.60–2.08	< LOD–8.90	< LOD–7.55
Enniatin B_1_	< LOQ	< LOD–1.22	< LOD–2.13	< LOD

### Hematological examination

The results of hematological examination are presented in Table 2.

**Table 2:** Hematological comparison of healthy (no visible disease signs, *n* = 30) and diseased (visible disease signs, *n* = 30) *Salmo trutta* m. *trutta* specimens from Słupia river. Mean parameter values are shown ± SD. Mean values with different superscripts between health status are significantly (*P* < 0.05) different

Variable	RBC (T/L)	HGB (g/dL)	PCV (%)	MCV (fL)	MCH (pg)	MCHC (g/dL)	WBC (k/μL)
Healthy	1.14^a^ ± 0.14	10.18^a^ ± 0.85	53.27^a^ ± 5.32	471.1^a^ ± 60.52	99.5^a^ ± 10.41	21.26^a^ ± 1.89	10.43^a^ ± 5.13
Diseased	1.24^b^ ± 0.36	9.6^b^ ± 2.5	44.97^b^ ± 10.86	385.61^b^ ± 110.92	83.12^b^ ± 26.06	21.52^a^ ± 1.55	4.49^b^ ± 2.8
Mann–Whitney *U* test result (*P* value)	− 2.28 (0.0215)	1.96 (0.0496)	3.58 (0.0002)	4.85 (0.0001)	4.83 (0.0001)	-	5.01 (0.0001)

*RBC*, red blood cell count; *HGB*, hemoglobin concentration; *PCV*, packed cell volume; *MCV*, mean corpuscular volume; *MCH*, mean corpuscular hemoglobin; *MCHC*, mean corpuscular hemoglobin concentration; *WBC*, white blood cell count

Statistically significant differences (*P <* 0.05) between the healthy and diseased fish were observed in six hematological parameters: RBC (*P* = 0.022), HGB (*P* = 0.049), PCV (*P* = 0.0002), MCV (*P* = 0.000001), MCH (*P* = 0.000001), and WBC (*P* = 0.000001). Compared with healthy specimens, higher RBC count and lower values of HGB, PCV, MCV, MCH, and WBC were observed in the diseased fish. There was no statistical difference (*P >* 0.05) in MCHC between fish with different health status.

## Discussion

In the recent year, prominent health disorders and mass mortality of brown trout spawners were noticed in many Polish rivers (data not shown) during the return of fish from the Baltic Sea to the river for spawning. In Slupia River, brown trouts with health disorders accounted about 70% of the spawning population. This river is located in the Pomeranian Landscape Park and considered to be a spawning place for brown trout, mainly due to the water parameters: the highest purity class, temperature not exceeding 10 °C in the summer, and good oxygenation. The observed clinical symptoms were suggestive of UDN-like syndrome, a disease of salmonids, the etiology of which remains unclear (Buller 2014). Various factors, including fungi from the genera *Saprolegnia* and *Aphanomyces*, have been taken into account as playing an essential role in the disease development. Those two fungal species, known as saprophytic opportunists, were considered to be either primary causative agents of UDN (Huxley 1882; Stirling 1881) or secondary ones to bacteria (Hume Patterson 1903) and viruses (Roberts 1972). However, there were also reports of ulcerative skin lesions caused by another fungus, *Fusarium solani*, described in two shark species: bonnethead shark (*Sphyrna tiburo*) (Muhvich et al. 1989) and hammerhead shark (*S*. *lewini*) (Crow et al. 1995). The same species of fungus together with *F*. *oxysporum* and *F*. *tabacinum* were responsible for black gill disease in shellfish such as prawn *Penaeus japonicus* (Khoa and Hatai 2005; Khoa et al. 2005) and Atlantic stream crayfish *Austropotamobius pallipes* (Alderman and Polglase 1985). Although recently a few reports regarding the diversity of fungi and mycotoxins in aquaculture have been published (Pietsch 2020; Viegas et al. 2019), there is no data linking the presence of *Fusarium* sp. and their mycotoxins in fish with UDN.

In the present study, the results of the toxicological analysis of the tissues obtained from the brown trout displaying the UDN-like symptoms, revealed zearalenone in elevated concentrations, mainly in the liver and kidneys. The levels of ZEN in the liver samples were comparable with the data obtained by Woźny et al. (2019) in the feeding trials involving rainbow trout fed with ZEN-containing feed (ZEN ~ 2 mg/kg). As a result of these trials, the concentration of ZEN in the liver was determined to be below 10 μg/kg of the tissue (Woźny et al. 2019). Regarding the mycotoxin detection in a fish muscle, similar to the present report, trace amounts of ZEN and its metabolites were noticed by other authors (Pietsch et al. 2015b) in carp. Overall, there are a low number of published data regarding ZEN concentration in tissues and its biotransformation in brown trout in comparison with other fish species (Malekinejad and Agh 2016).

Considering that the concentrations of the mycotoxins in the tissues in the present study were low, they were not likely to be the leading cause of the fish mortality. However, their detection supported by the results of mycological examination seems to confirm *Fusarium* infection. Isolation of the fungus hyphae followed by their identification as *Fusarium* spp. indicated a relationship between the occurrence of the mycotoxins and the presence of the fungus in fish. Apart from the fungi, there were bacteria isolated from the tissues. It might suggest their involvement in the pathology of the disease, including development of the skin lesions. However, despite the various microbial species isolated from the brown trout, all of them are known as opportunistic pathogens for fish (Austin and Austin 2016). Therefore, in this case, the role of bacteria in the development of the health disorders was pointed out a secondary one, which was also previously described (Hume Patterson 1903). There were no viruses detected in the examined fish tissues.

Microscopic skin lesions, particularly the loss of epithelium, dermal necrosis, and tissue disruption by the fungal hyphae, were consistent with changes associated with fusariomycosis described by other authors (Naples et al. 2012; Salter et al. 2012). Among the examined brown trout in the present study, there were only single cases in which the inflammatory cells were visible in the tissue. These findings differ from the reports by certain authors who noted granulomatous inflammation associated with *Fusarium* spp*.*, i.e., *Fusarium solani* in hammerhead sharks (Desoubeaux et al. 2018; Pirarat et al. 2016) or *Fusarium oxysporum* in zebrafish (Kulatunga et al. 2017) and in tilapia (Cutuli et al. 2015). Internal organs in the examined cases were not affected, similar to reports by Salter et al. (2012). Therefore, the initial fungal infection in these cases seems to be external, progressing from the epithelium to deeper layers, reaching the blood vessels which spread mycotoxins to other organs. While other authors have previously described this route of *Fusarium* infection (Guarner and Brandt 2011), the inside-to-outside fungal dissemination was also reported (Cutuli et al. 2015). The available reports of *Fusarium* mycoses causing ulcerative integument lesions in fish concern mostly such species as *F*. *solani* or *F*. *oxysporum* (Abd El-Ghany et al. 2014; Cutuli et al. 2015; Desoubeaux et al. 2018; Salter et al. 2012) whereas *F*. *avenaceum* has been reported as the main pathogen associated with shell erosions in crayfish (Makkonen et al. 2013).

The results of hematological examinations revealed disturbances in the general state of fish health. The main reason for this seems to be the skin damage because an integrity of the integument plays an essential role in maintaining fish homeostasis by preventing water intake and osmotic stress (Noga 2000). The skin disruption associated with fungal invasion might be responsible for osmoregulation failure. The decrease in HGB, PCV, MCV, MCH, and WBC levels, similar to the previous reports (Cieplinski et al. 2018), was most likely the result of displacement of the water from the tissues into the bloodstream in response to osmotic stress. Very low WBC values observed among samples collected from the diseased brown trout indicate severe immunosuppression.

The results of the present study reveal a potential relationship between the invasion of *Fusarium* sp. in brown trout and the presence of mycotoxins in the internal organs of the fish. To the authors’ knowledge, this is the first report of *Fusarium* sp. infection in brown trout, taking into account an aspect of ZEN toxicity. Moreover, the results of the brown trout skin analysis provide new information with regard to determination of UDN etiology. The question about the causes of these fungus invasions among the wild population of brown trout remains open, and it will be the subject of our further investigations.

## Electronic supplementary material

ESM 1 (DOCX 15 kb)

## References

[CR1] Abd El-Ghany NA, El-Khatib NR, Salama SSA (2014). Causes of mortality in discus fish (*Symphysodon*) and trials for treatment. Egypt J Aquac.

[CR2] Alderman DJ, Polglase JL (1985). *Fusarium tabacinum* (Beyma) Gams, as a gill parasite in the cray-fish, *Austropotamobius pallipes* Lereboullet. J Fish Dis.

[CR3] Austin B, Austin DA (2016) Bacterial fish pathogens. Disease of farmed and wild fish. 6th edition. Springer International Publishing Switzerland

[CR4] Buller NB (2014). Bacteria and fungi from fish and other aquatic animals: a practical identification manual.

[CR5] Chełkowski J (1985). Mikotoksyny, wytwarzające je grzyby i mikotoksynozy.

[CR6] Cieplinski M, Kasprzak M, Grandtke M, Steliga A, Kaminski P, Jerzak L (2018). The effect of dipotassium EDTA and lithium heparin on hematologic values of farmed brown trout *Salmo trutta* (L.) spawners. Aquac Int.

[CR7] Crow GL, Brock JA, Kaiser S (1995). *Fusarium solani* fungal infection of the lateral line canal system in captive scalloped hammerhead sharks (*Sphyrna lewini*) in Hawaii. J Wildl Dis.

[CR8] Cutuli MT, Gibello A, Rodriguez-Bertos A, Blanco MM, Villarroel M, Giraldo A, Guarro J (2015). Skin and subcutaneous mycoses in tilapia (*Oreochromis niloticus*) caused by *Fusarium oxysporum* in coinfection with *Aeromonas hydrophila*. Med Mycol Case Rep.

[CR9] Desoubeaux G, Debourgogne A, Wiederhold NP, Zaffino M, Sutton D, Burns RE, Frasca S, Hyatt MW, Cray C (2018). Multi-locus sequence typing provides epidemiological insights for diseased sharks infected with fungi belonging to the *Fusarium solani* species complex. Med Mycol.

[CR10] Ferrer C, Colom F, Frases S, Mulet E, Abad JL, Alio AL (2001) Detection and identification of fungal pathogens by PCR and by ITS2and 5.8S ribosomal DNA typing in ocular infections. J. Clin. Microbiol 39, 2873–2879. 10.1128/JCM.39.8.2873-2879 10.1128/JCM.39.8.2873-2879.2001 PMC88253 11474006

[CR11] Fiedler K, Schutz E, Geh S (2001). Detection of microbial volatile organic compounds (MVOCs) produced by moulds on various materials. Int J Hyg Environ Health.

[CR12] Guarner J, Brandt ME (2011). Histopathologic diagnosis of fungal infections in the 21st century. Clin Microbiol Rev.

[CR13] Gupta AK, Baran R, Summerbell RC (2000). *Fusarium* infections of the skin. Curr Opin Infect Dis.

[CR14] Hume Patterson J (1903) On the cause of salmon disease, a bacteriological investigation. Fisheries Board of Scotland and Salmon Fisheries Cd. 1544, HMSO, Glasgow, 55

[CR15] Huxley TH (1882). A contribution to the pathology or the epidemic known as the salmon disease. Proc R Soc.

[CR16] Jeschke N, Nelson PE, Marasas WFO (1990). *Fusarium* species isolated from soil samples collected at different altitudes in Transkei, Southern Africa. Mycologia.

[CR17] Khoa LV, Hatai K (2005). First case of *Fusarium oxysporum* infection in cultured kuruma prawn *Penaeus japonicus* in Japan. Fish Pathol.

[CR18] Khoa LV, Hatai K, Yuasa A, Sawada K (2005). Morphology and molecular phylogeny of *Fusarium solani* isolated from kuruma prawn *Penaeus japonicus* with black gills. Fish Pathol.

[CR19] Kulatunga DC, Dananjaya SH, Park BK, Kim CH, Lee J, De Zoysa M (2017). First report of *Fusarium oxysporum* species complex infection in zebrafish culturing system. J Fish Dis.

[CR20] Makkonen J, Jussila J, Koistinen L, Paaver T, Hurt M, Kokko HJ (2013). *Fusarium avenaceum* causes burn spot disease syndrome in noble crayfish (*Astacus astacus*). J Invertebr Pathol.

[CR21] Malekinejad H, Agh N (2016). Interspecies variation in the hepatic biotransformation of zearalenone: evidence for bio-inactivation of mycoestrogen zearalenone in sturgeon fish. Iran J Fish Sci.

[CR22] Muhvich AG, Reimschuessel R, Lipsky MM, Benneti RO (1989). *Fusarium solani* isolated from newborn bonnethead sharks, *Sphyrna tiburo* (L). J Fish Dis.

[CR23] Murrphy T (1973). Ulcerative dermal necrosis (UDN) of salmonid - a review. Ir Vet J.

[CR24] Naples LM, Poll CP, Berzins IK (2012). Successful treatment of a severe case of fusariomycosis in a beluga whale (*Delphinapterus leucas leucas*). J Zoo Wildl Med.

[CR25] Noga ED (2000). Skin ulcers in fish: *Pfiesteria* and other etiologies. Toxicol Pathol.

[CR26] Panasiuk L, Jedziniak P, Pietruszka K, Piatkowska M, Bocian L (2019). Frequency and levels of regulated and emerging mycotoxins in silage in Poland. Mycotoxin Res.

[CR27] Pękala A, Paździor E, Antychowicz J, Bernad A, Głowacka H, Więcek B, Niemczuk W (2018). *Kocuria rhizophila* and *Micrococcus luteus* as emerging opportunist pathogens in brown trout (*Salmo trutta* Linnaeus, 1758) and rainbow trout (*Oncorhynchus mykiss* Walbaum, 1792). Aquaculture.

[CR28] Pietsch C (2020). Risk assessment for mycotoxin contamination in fish feeds in Europe. Mycotoxin Res.

[CR29] Pietsch C, Katzenback BA, Garcia-Garcia E, Schulz C, Belosevic M, Burkhardt-Holm P (2015). Acute and subchronic effects on immune responses of carp (*Cyprinus carpio* L.) after exposure to deoxynivalenol (DON) in feed. Mycotoxin Res.

[CR30] Pietsch C, Kersten S, Burkhardt-Holm P, Valenta H, Dänicke S (2013). Occurrence of deoxynivalenol and zearalenone in commercial fish feed: an initial study. Toxins (Basel).

[CR31] Pietsch C, Kersten S, Valenta H, Dänicke S, Schulz C, Burkhardt-Holm P, Junge R (2015). Effects of dietary exposure to zearalenone (ZEN) on carp (*Cyprinus carpio* L.). Toxins (Basel).

[CR32] Pirarat N, Sahatrakul K, Lacharoje S, Lombardini E, Chansue N, Techangamsuwan S (2016). Molecular and pathological characterization of *Fusarium solani* species complex infection in the head and lateral line system of Sphyrna lewini. Dis Aquat Org.

[CR33] Roberts RJ (1972). Ulcerative dermal necrosis (UDN) of salmon (*Salmon salar* L). Symp Zool Soc London.

[CR34] Roberts RJ, Ball HH, Munro ALS, Shearer WM (1971). Studies of ulcerative dermal necrosis of salmonids III The healing process in fish maintained under experimental condition. J Fish Biol.

[CR35] Salter CE, O’Donnell K, Sutton DA, Marancik DP, Knowles S, Clauss TM, Berliner AL, Camus AC (2012). Dermatitis and systemic mycosis in lined seahorses *Hippocampus erectus* associated with a marine-adapted *Fusarium solani* species complex pathogen. Dis Aquat Org.

[CR36] Schollenberger M, Drochner W, Muller HM (2007). *Fusarium* toxins of the scirpentriol subgroup: a review. Mycopathologia.

[CR37] StatSoft (2006) Elektroniczny Podręcznik Statystyki PL. Krakow

[CR38] Willoughby LG (1989). Continued defence of salmonid fish against *Saprolegni*a fungus, after its establishment. J Fish Dis.

[CR39] Woźny M, Obremski K, Hliwa P, Gomułka P, Różyński R, Wojtacha P, Florczyk M, Segner H, Brzuzan P (2019). Feed contamination with zearalenone promotes growth but affects the immune system of rainbow trout. Fish Shellfish Immunol.

[CR40] Viegas C, Esteves L, Faria T, Pombo A, Caetano LA, Quintal-Gomes A, Twarużek M, Kosicki R, Gajewski J, Viegas S (2019). Fungal diversity and mycotoxin distribution in echinoderm aquaculture. Mycotoxin Res.

