# Supplementary material for: Could mycotoxigenic Fusarium sp. play a role in ulcerative dermal necrosis (UDN) of brown trout (Salmo trutta morpha trutta)?
Source: Mycotoxin Res. 2020 May 5;36(3):311–8. doi: 10.1007/s12550-020-00395-8 (PMC7359170; doi:10.1007/s12550-020-00395-8)
Supplement: Supplementary file 1 — (DOCX 15 kb) [file 12550_2020_395_MOESM1_ESM.docx]

LOD and LOQ values for mycotoxin determination in fish tissues by LC-MS/MS

| Analyte | LOD (µg/kg) | LOQ (µg/kg) |
| --- | --- | --- |
| aflatoxin B_1_ | 1.0 | 2.5 |
| aflatoxin B_2_ | 1.0 | 2.5 |
| aflatoxin G_1_ | 1.0 | 2.5 |
| aflatoxin G_2_ | 1.0 | 2.5 |
| alfa-zearalanon | 0.5 | 1.0 |
| beauvericin | 0.05 | 0.1 |
| beta zearalanol | 0.5 | 1.0 |
| citrinine | 2.0 | 5.0 |
| deoxynivalenol | 5.0 | 15.0 |
| diacetoxyscirpenol | 5.0 | 10.0 |
| enniatin A | 0.05 | 0.1 |
| enniatin A_1_ | 0.05 | 0.1 |
| enniatin B | 0.05 | 0.1 |
| enniatin B_1_ | 0.05 | 0.1 |
| fumonisin B_1_ | 5.0 | 10.0 |
| fumonisin B_2_ | 5.0 | 10.0 |
| fusarenon-X | 5.0 | 10.0 |
| HT-2 | 10.0 | 25.0 |
| nivalenol | 5.0 | 10.0 |
| ochratoxin A | 2.0 | 5.0 |
| sterigmatocystin | 0.5 | 1.0 |
| T-2 | 5.0 | 10.0 |
| Zearalenon | 0.5 | 1.0 |
| zearalenone | 0.5 | 1.0 |
| α - zearalanol | 0.5 | 1.0 |
| β - zearalenol | 0.5 | 1.0 |
